# Age and Sex in Back Pain Intensity—Retrospective Study of Conservatory vs. Surgical Discopathy Treatment

**DOI:** 10.3390/life12111808

**Published:** 2022-11-07

**Authors:** Kamil Koszela, Marta Woldańska-Okońska

**Affiliations:** 1 Neuroorthopedics and Neurology Clinic and Polyclinic, National Institute of Geriatrics, Rheumatology and Rehabilitation, 02-637 Warsaw, Poland; 2 Department of Rehabilitation and Physical Medicine, Medical University, 90-700 Lodz, Poland

**Keywords:** LBP, low back pain, gender, spine surgery, spine

## Abstract

(1) Background: The frequency of back pain diagnosis and treatment has markedly increased in recent years. Back pain may be caused by many factors and discopathy is one of them. The aim of the study was to assess the impact of age and sex on back pain intensity in rehabilitated patients with discopathy treated conservatively and surgically; (2) Methods: The study included 137 patients: 96 undergoing conservative therapy and 41 after back surgery due to discopathy. VAS and the Laitinen scale were used for pain assessment. All patients underwent a multidirectional rehabilitation program at the Department of Rehabilitation and Physical Medicine of the Medical University in Łódź; (3) Results: No statistically significant effect of age and sex was observed on the level of pain intensity on VAS and the Laitinen scale; (4) Conclusions: Age and sex do not seem to affect back pain intensity in rehabilitated patients treated conservatively and surgically for discopathy. The problem requires further research on a larger group of patients.

## 1. Introduction

The prevalence and treatment of back pain have increased significantly in recent years. Back pain and dysfunction syndrome affect more and more individuals and are considered to be associated with lifestyle factors. The lack of exercise, reduced physical activity, and non-physiological body posture, i.e., a negligent and too frequently assumed sitting position, are a strain on the lumbosacral spine and result in early degeneration [1].

Low back pain (LBP) is one of the most common complaints reported by patients [2]. It affects about 50–80% of the population at some point during life [3,4]. 

However, this percentage may be underestimated considering different levels of pain tolerance. Furthermore, it constitutes economic hardship for the budget, taking into account the cost of hospitalization and medical procedures, incapacity for work, and thus absence at work.

Non-specific low back pain (NLBP) is a frequent complication. NLBP has become a major public health problem worldwide. In total, 11–12% of the population is disabled by LBP [4,5]. Moreover, chronic pain is a maladaptive condition affecting 7–10% of the population worldwide and can be accompanied by anxiety, insomnia, and depression [6].

Mechanobiology helps to better elucidate the pathophysiology of the intervertebral disc (IVD) and its potential for biological repair. IVD is the largest avascular element in the human body and has a small number of cells. The unfavorable microenvironment is not conducive to bearing high levels of mechanical stress. Hydrostatic pressure strongly affects the nuclear pulposus (NP), whereas the fibrous ring (AF) is able to withstand biaxial tensile and shear forces. The cells of the nucleus pulposus and fibrous ring are exposed to high mechanical stresses due to body weight, voluntary and spontaneous activity, and vertebral stabilization forces. These cells are adapted to anaerobic metabolism with low oxygen pressure and acidic pH. The adaptation of cells to states of acidosis and increased osmotic pressure is regulated by mechanoproteins. They are responsible for the transmission of a mechanical signal to a cellular response [7].

Chronic oxygen and glucose deficiencies have a detrimental effect on cell viability. Oxygen deficiency causes cells to become inactive, while glucose deficiency can cause cell death. The hypoxic and acidotic disc cells as well as disc herniation cells are still biologically active and produce spontaneously nitric oxide; prostaglandin E2 (PGE2); interleukin-6 (IL-6); meta-protease ECM; and pro-inflammatory cytokines: including interleukin-1α (IL-1α), interleukin-1β (IL-1β), interleukin-6 (IL-6), tumor necrosis factor-alpha (TNF- α), and factors stimulating the accumulation of granulocytes and macrophages. On the one hand, these factors intensify the inflammatory process and pain, and on the other, they stimulate repair processes [7].

Back pain may be caused by many factors, one of which is discopathy. Discopathy is caused by a three-stage process of discosis. A degenerative disc disease is characterized by bulging, dislocation, and pressure on the nervous structures. The first stage of discosis is an early stage in adolescence and early adulthood and begins with the regression of intradiscal vessels. The disc tissue receives nutrients only by diffusion. The lower body weight of children and their vigorous physical activity generate advantageous biomechanics for the nourishment of the intervertebral disc fiber-producing cells. With the body weight increase, the axial load on the disc also increases, particularly in the lower lumbar region. As children grow up, they become less active, spending much time desk-bound at school and then in adulthood at work [8]. 

The second stage affects middle-aged adults. The intervertebral disc may be displaced, and therapeutic procedures must be applied because, in this stage, the disc produces intense local and radicular pain. Protrusion, herniation, and sequestration, which can compress nerve roots, are observed [8,9].

The third stage concerns the old age population. The disc tissue loses water, the disc becomes thinner and does not absorb shocks, it becomes fibrotic and firm. Further calcification of the ligaments results in limited segmental mobility [8]. 

Studies have shown that degenerative changes within intervertebral discs are observed in the population of subjects over 30 years of age, and the incidence of spondylosis increases with age and reaches 100% in persons aged over 80–90 years [8].

These problems require adequate clinical and imaging diagnostics in order to implement appropriate management. It is important to identify the cause of symptoms and introduce appropriate causal treatment rather than focus only on symptomatic therapy. 

A patient with back pain initially reports to a primary care physician. This is usually the beginning of the diagnostic process. In addition to the clinical examination of the patient, it is important to perform imaging diagnostics of the spine: X-ray, computed tomography (CT), or magnetic resonance imaging (MRI). MRI is the gold standard in diagnosing intervertebral disc diseases. Moreover, the correlation of changes in the radiological image with clinical symptoms is of great importance. In the first place, the diagnostic process should be based on conservative methods (such as changes in lifestyle, pharmacotherapy, and rehabilitation). Kinesitherapy is very important in the rehabilitation process. It improves circulation within peri-spinal soft tissues, causes relaxation and stretching, as well as strengthens soft tissues. Surgical treatment is recommended only when a lack of improvement is observed. However, there is always a risk of failed back surgery syndrome (FBSS). FBSS consists of three issues: 1. Instability associated with the surgery, 2. Patient’s predispositions. 3. Tissue scarring [1,8].

The problem of the diagnosis and treatment of back pain is of great significance. Early diagnosis and treatment minimize the cost and reduce the risk of spine surgery, which is expensive compared to conservative management. Therefore, patients should be under multidisciplinary care [10].

Due to the social scope of diseases of the spine, the dimension of their distribution among different age groups and sexes is important. The meta-analysis of Souza IMB et al. [11] included over 2000 studies and valued 135,059 elderly people, aged 60 to 102 years; the incidence of LBP ranged from 21% to 75%. The levels of disability as well as functional difficulties and problems with activities of daily living and physical performance were found in 60% of the investigated subjects. In Hong Kong, a large prospective skeletal system health study was conducted on elderly people (≥65 years old) including 2000 men and 2000 women by examining disc space narrowing (DSN). The height of the L1/L2–L4/L5 intervertebral space was assessed on lateral radiographs of the lumbar region. The study revealed that the difference between men and women in DSN severity increased with age. Recent evidence suggests that estrogen, through various potential biological effects, also seems to affect disc degeneration in postmenopausal women. 

A large-scale population study shows that with age, disc space narrowing progresses faster in women than in men. Significant disc space narrowing is associated with higher bone mineral density BMD of the spine and hips, back pain, and reduced leg mobility [12].

The obtained results do not indicate the level of pain and disability. In contrast, in another study in China [13], the incidence—years lived with disability (YLD) and low back pain—decreased slightly from 1990 to 2016. However, the total number of LBP and YLD sufferers increased (due to the increased country population). Back pain continues to be the second leading cause of YLD-related diseases. LBP is still a global problem, especially in the female population.

Quite numerous reports indicate that the incidence of low back pain may be related to psychosocial risk factors in the workplace [14]. Moreover, low back pain often seems to be part of a widespread pain problem, rather than an isolated, regional pain [15]. There is an association of low back pain and exposure to a hostile working environment, job insecurity, long working hours, and certain occupational groups. These factors should be taken into account by employers, policymakers, and health workers who are concerned about the impact of low back pain on the health and social consequences of workers [16]. The sex and age correlations may affect broadly understood prevention programs related to this problem.

The aim of the study was to assess the impact of age and sex on back pain intensity in rehabilitated patients with discopathy treated conservatively or surgically.

Hypothesis: Age and gender do not affect back pain intensity in rehabilitated patients treated surgically and conservatively for discopathy.

## 2. Materials and Methods

### 2.1. Study Population

The study included 137 subjects aged 19–89 years (mean 62 years, SD 12.11): 77 women and 60 men (men constituted 44%), hospitalized at the Department of Rehabilitation and Physical Medicine of the Medical University of Lodz (1 January 2014–31 December 2016) due to back pain. The patients reported back pain due to discopathy, i.e., protrusion, herniated nucleus pulposus, and sequestration. The complaints were confirmed by spine MRI and CT. On admission, the patients were examined, and the medical history was collected. Twenty-one percent (*n* = 29) of hospitalized patients smoked cigarettes, including nine surgically treated.

Each patient gave written consent for hospitalization, examination, and treatment.

This study was conducted according to the guidelines of the Declaration of Helsinki and was approved by the Bioethics Committee for Scientific Research at the Medical University of Lodz, Poland (No: RNN/208/16/KE).

Inclusion criteria:

- back pain syndrome caused by discopathy and confirmed by MRI or CT,
- chronic pain (>3 months),
- the patient’s consent to the suggested treatment and rehabilitation presented in the medical history,
- no contraindications for physiotherapy and kinesitherapy.
- Exclusion criteria
- back pain syndrome without discopathy on MRI or CT,
- active neoplastic disease, spondyloarthropathies, and other serious diseases;
- lack of the patient’s consent to the proposed rehabilitation,
- contraindications to the proposed rehabilitation.

### 2.2. Study Protocol

Prior to admission, some patients (*n* = 96) were treated only with conservative methods. The remaining patients (*n* = 41) underwent spinal surgery due to back pain in the course of discopathy. Twenty patients (49%) underwent fusion surgery: PLIF (Posterior Lumbar Interbody Fusion) or ACIF (Anterior Cervical Interbody Fusion). The other 21 (51%) underwent surgery involving the removal of a herniated disc or a sequester without fusion. These were microdiscectomies and discectomies [8].

After admission, all patients (*n* = 137) were qualified for treatment with kinesi- and physical therapy for 6 days a week. Kinesitherapy was based on isometric exercises, active load-free, balance, and loosening, as well as relaxation exercises. The McKenzie and NDT-Bobath methods were also used. Moreover, gait training exercises were applied. Kinesitherapy lasted approximately 2.5 h a day. It included, among others, classical kinesitherapy, exercises using the Bobath method, and exercises with biofeedback on a balance platform. Physical therapy was based on the application of magnetic field, laser, and electrotherapy. The treatment regimen was the same in both groups. Rehabilitation was carried out by the same rehabilitation team.

The Visual Analogue and Laitinen scales were used to analyze pain intensity and frequency on admission and on discharge. Furthermore, the use of analgesics and limitation in motor activity were investigated for the clinical assessment of the level of spinal pain. The maximum value on the Laitinen scale was 16 and the minimum was 0 [17].

The average hospitalization period was 29 days (±SD 5.77).

All rehabilitation procedures were supervised by one physician. No complications were observed during the rehabilitation cycle. All patients were discharged in good general condition and referred for further outpatient treatment. 

### 2.3. Data Analysis

Continuous and ordinary data were presented as median and interquartile ranges. The Shapiro–Wilk test was used to test the normality of the distribution in each group. Spearman’s rank correlation coefficient was also used to evaluate the relationship between age and change in the scale score. The Mann–Whitney test was used for group comparisons. All statistical analyses were carried out with the use of STATISTICA 13.1 (TIBCO Software, Palo Alto, CA, USA). A *p*-value < 0.05 was considered statistically significant. 

## 3. Results

The presented tables and figures demonstrate the results of the effect of age and sex on the level of pain intensity in the VAS and Laitinen scales in the group of patients with back pain treated surgically or non-surgically due to discopathy. 

Table 1 presents participant characteristics. There were no statistically significant differences in the studied groups.

Table 2 presents the influence of sex on the difference in the VAS and Laitinen scales in the non-operated and operated groups. There were no statistically significant differences in the studied groups.

**Figure 1:**
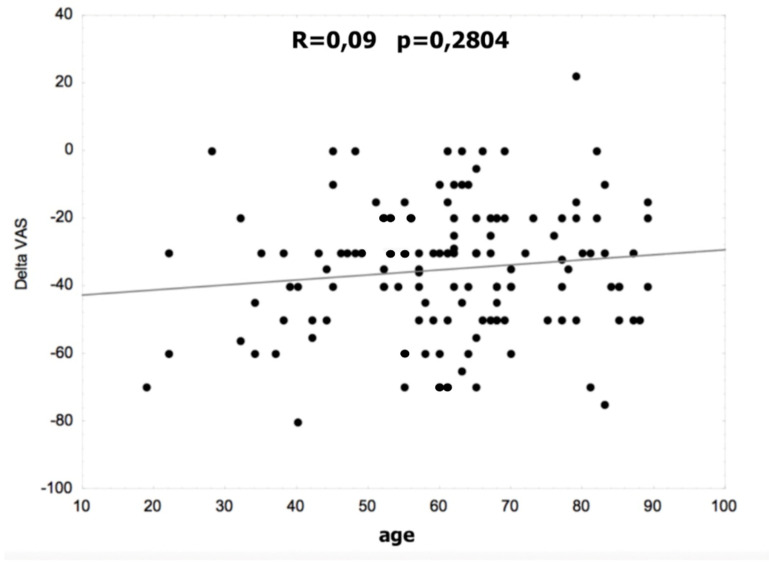
Correlation between age and the difference in VAS.

**Figure 2:**
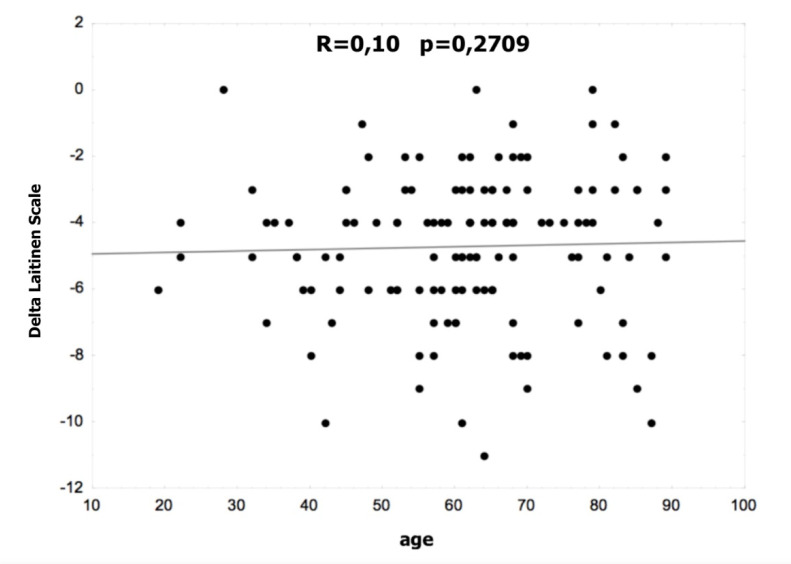
Correlation between age and the difference in the Laitinen scale.

Figure 1 and Figure 2 show the correlation between age and the difference in the VAS and Laitinen scales. There were no statistically significant differences in the non-operated and operated groups.

Table 3 presents the influence of rehabilitation on the VAS and Laitinen scales in the non-operated and operated groups. There were statistically significant differences in the studied groups.

## 4. Discussion

Back pain syndrome affects a very large social group. International reports present precise data on the incidence of spine pathologies, including those of intervertebral discs. The recognition of the causes of ailments, the implementation of proper causal treatment, and not focusing only on the symptomatic treatment are of great importance.

Back pain can originate from three different sources. These are axial lumbosacral, radicular, and transferred pain. The annual prevalence of low back pain in the US adult population is 10–30%, and the lifetime prevalence is as high as 65–80% [8].

The patient’s medical history, physical and functional examination, and diagnostic tests are important elements of accurate diagnosis and identification of the patient’s pathophysiology. There are many types of back pain: myofascial, intervertebral joint, sacroiliac joint, discogenic, spinal stenosis, and failed back surgery pain. Patients with chronic back pain require a multidisciplinary, patient-centered treatment that should include multimodal medical, psychological, physical, and interventional approaches. Back pain is a complex and difficult-to-treat therapeutic problem that affects millions of Americans every year [18]. In the presented results, we observed a significant improvement after the implemented rehabilitation in non-operated and operated patients. However, the Delta on the Laitinen and VAS scales shows a tendency for a greater (although not statistically demonstrated) improvement in the group of non-operated patients, which would be worth verifying in a larger group of patients. 

The possible differences in the outcomes of LBP therapy between women and men can be determined by biological differences in immune function that may underlie these observed effects. Women far outnumber men as sufferers of chronic pain syndromes. However, social and psychological factors also play a role in the prevalence of chronic pain in both sexes. These differences can be attributed, among others, to diversity in cell populations (T lymphocytes), differences in hormone suppression, and different cellular responses in males and females. These sex differences also affect human cellular responses and may be the mechanism by which disproportionate chronic pain is demonstrated. Identifying the path of mechanisms underlying sex differences in pain may be a clue for therapy and provide better options for patients [19].

The studies of Canadian scientists indicated the sex-dependent activation of brain centers in chronic pain of inflammatory nature—ankylosing spondylitis. Females showed higher functional segregation in the medial cingulate cortex and anterior cingulate cortex, and lower network connectivity with the default mode and frontoparietal modules, while males showed stronger connectivity with the sensorimotor module. On the basis of these observations, it was possible to determine whether a given person suffered from chronic pain and even what gender he/she was with an accuracy of 77–92%. These findings highlight the organizational abnormalities of the neural network in the resting state of the brain in people with chronic pain and are the basis for the production of sex-specific analgesics in the future [20].

Some sources informed about the impact of age on the susceptibility to spine diseases; age over 50 was treated as a risk factor, most likely related to pain caused by degenerative spine disease. Moreover, a correlation was seen between the spine pain syndrome and the symptoms of accelerated brain aging [21]. Differences in factors associated with musculoskeletal pain in older age were also reported [22]. The pain was correlated with fair or poor self-rated health, a history of back pain before the age of 65, and co-existing disability both in men and women. Body mass index, systolic blood pressure, and depressive symptoms were factors associated with pain only in women. In men, pain was related to polyarticular radiographic osteoarthritis, including that of the spine. Thus, both sexes differ in factors associated with musculoskeletal pain in old age. Finally, it might be concluded that musculoskeletal pain was more frequent in older women than in older men. These observations were reported in the 22-year Framingham Study (1992–1993), which included 682 women and 380 men aged 72 years and older.

On the other hand, in a large study of 202,077 men and 237,754 women in a UK bio-bank, the genetic factor of chronic back pain shows a mild sex and age relationship [23]. The genetic correlation between the sex decreased with age. There was also a stronger genetic correlation with the diagnosis of disc degeneration in men than in women. The above-described correlations were not observed in this study. 

A systematic review of clinical trials programmed to the risk of chronic disease in adults with acute or subacute musculoskeletal pain was conducted by C. Meyer et al. and showed moderate results [24]. The rehabilitation program was used to introduce simple educational messages that were sufficient for low-risk patients. Moderate-to-high-risk patients benefited from a physical rehabilitation program combined with education. In high-risk patients, an additional cognitive-behavioral intervention improved the outcome even more.

It is possible that the cause of low back pain is significantly influenced by patients’ sex representation. In the studies of Tamartash H. et al., various hypotheses concerning the role of connective tissue of the thoracolumbar fascia TLF in low back pain were presented. The changes were examined on USG bilaterally at levels L2–L3 and L4–L5 in both the group of patients with non-specific LBP and the control group without pain. The examined parts of the fascia were located 2 cm to the side of the center of the interspinal ligament. There were no significant differences in age, sex, and BMI between LBP and healthy subjects. There was also no significant relationship between age, BMI, and sex with the TLF elasticity index. There was a strong inverse relationship between pain intensity (*p* = 0.004) and the TLF elasticity index. Patients with LBP had a 25–30% lower TLF modulus of elasticity than healthy subjects [25].

It is worth noting that large studies analyzing numerous studies or a large number of patients do not differentiate in detail the etiology of low back pain. Perhaps the pain of non-nervous etiology has a stronger impact on its perception in the male sex. However, in the above study, patients with neuralgia-type pain were described. In the analysis of the intensity of pain in women and men with LBP, its intensity should be analyzed depending on the etiology of the pain [25].

Although rehabilitation, as shown in the above study, gives good results in both postoperative and conservative treatment, its effects are not permanent. Although no correlation was found in the above study related to the LBP and age, it is worth noting that the average age of rehabilitated patients was 62 years. Moreover, another pain incident could make patients more disabled.

More research is needed to understand the sex differences in musculoskeletal pain in the older population.

The obtained results prove that further analyses are required, supplemented with long-term follow-up in a larger group of patients. The presented study is retrospective. Thus, it is possible to analyze the results obtained much earlier without the possibility of introducing any corrections to the protocol. Therefore, it is worth conducting a prospective study. It would be worthwhile to conduct another prospective study enriched by the pain assessment while testing muscle tension or by examining inflammatory parameters such as interleukin1α (IL-1α), interleukin-1β (IL-1β), interleukin-6 (IL-6), tumor necrosis factor-alpha (TNF-α), and the observation of their changes during treatment and rehabilitation.

## 5. Conclusions

Age and sex did not affect back pain intensity in rehabilitated patients treated conservatively and surgically for discopathy. However, imaging tests proved that pain does not always correlate with the severity of the degenerative process. Further research is required in a larger group of patients taking into account both the clinical and radiological picture of the disease and subjective disorders.

## Figures and Tables

**Table 1:** Participant characteristics.

	Median (IQR)	Non-Operated Group (*n* = 96)	Operated Group (*n* = 41)	*p*
		Median (IQR)	Median (IQR)	
Age [year]	63.00 (53.00–70.00)	63.5 (54.5–76)	61.00 (49.00–67.00)	0.2154
Height [m]	1.68 (1.63–1.75)	1.68 (1.63–1.75)	1.68 (1.64–1.74)	0.9850
Weight [kg]	78.00 (68.00–90.00)	75.00 (70.00–84.00)	85.00 (65.00–91.00)	0.2291
BMI [kg/m^2^]	27.26 (24.44–30.11)	26.35 (24.15–28.41)	29.37 (26.03–30.92)	0.2046
	*n* (%)	Non-operated group (*n* = 96)	Operated group (*n* = 41)	*p*
		*n* (%)	*n* (%)	
Men	60 (43.80%)	41 (42.71%)	19 (46.34%)	0.6947

IQR—the interquartile range, *p*—level of significance, *n*—number.

**Table 2:** Influence of sex on the difference in the Laitinen scale and the VAS scale in the studied groups.

	Non-Operated Group (*n* = 96)		
	Women	Men	*p*
Delta VAS median (IQR)	−35.0 (−50.0 to −20.0)	−40.0 (−50.0 to −30.0)	0.5461
Delta Laitinen Scale median (IQR)	−5.0 (−7.0 to −4.0)	−5.0 (−6.0 to −3.0)	0.4620
	Operated group (*n* = 41)		
	women	men	*p*
Delta VAS median (IQR)	−30.0(−40.0 to −15.0)	−30.0(−50.0 to −20.0)	0.4524
Delta Laitinen Scale median (IQR)	−4.0 (−4.0 to −3.0)	−4.0 (−5.0 to −3.0)	0.9889

IQR—the interquartile range, *p*—level of significance, *n*—number.

**Table 3:** Laitinen and VAS score assessment before and after rehabilitation in the non-operated and operated groups.

	Non-Operated Group (*n* = 96)			
	Before Rehabilitation Median (IQR)	After Rehabilitation Median (IQR)	Delta Median (IQR)	*p*
VAS	70.0 (60.0–80.0)	30.0 (20.0–40.0)	−40.0 (−50.0–−25.0)	0.0001
Laitinen Scale	8.0 (7.0–10.0)	3.0 (3.0–5.0)	−5.0 (−6.0–−4.0)	0.0001
	Operated Group (*n* = 41)			
	Before rehabilitation median (IQR)	After rehabilitation median (IQR)	Delta median (IQR)	*p*
VAS	70.0 (48.5–80.0)	30.0 (15.0–40.0)	−30.0 (−45.0–−17.50)	0.0001
Laitinen Scale	8.0 (7.0–10.0)	4.0 (3.0–5.0)	−4.0 (−5.0–−3.0)	0.0001

## Data Availability

The data is available from the corresponding author if required.
